# A New Champion of Medical Women's Rights

**Published:** 1890-11-22

**Authors:** 


### The Doctor's Armchair.

A NEW CHAMPION OF MEDICAL WOMEN'S RIGHTS.

Sir Henry Acland has earned the gratitude of women, and at the same time of reasonable men, by his letter to the *Times* on the "Admission of women to examinations in medicine at Oxford." Lovers of plain sense and of solid intellectual progress will thank Professor Case, too, for taking up cudgels on behalf of a feeble and now happily decaying conventionalism. A good cause has little to lose and much to gain by opposition. In the present instance the good cause has the advantage of a strong champion: the bad cause is headed by Professor Case; and though for the moment he has won barren victory, we do not desire for it another head. By way of adding a little piquancy to our opinion of Professor Case's fitness for leading the tattered and dilapidated remnants of the army of effete conventionalism, we may mention a little incident which happened about a year ago. Professor Case was then rallying a detachment of his army against some other advancing movement; and the exhibition he made of himself in one of the morning papers was so very peculiar that a London editor who read his letter came to the conclusion that he must be still cutting his milk teeth. Whereupon, by way of offering to the distinguished Professor a present that would be both acceptable and useful, he purchased an improved baby's feeding bottle, and ordered it to be packed and addressed to the Professor's residence at Oxford. The bottle was never sent, because the donor's heart relented before the dispatch of the mail. He reflected that though Mr. Case was an exceedingly peculiar person, he was still an Oxford Professor; and he thought that though little might be due to the man, much was due to the Professor. Mr. Case will do well to consider that a man of position cannot be foolish in a morning newspaper without his folly being remembered by other people although it may be forgotten by himself.

Mr. Case opposes, and Sir Henry Acland supports the claims of women to be admitted to examinations in medicine at Oxford. Oxford is a great University and must act in its corporate capacity with a judgment and gravity befitting its character. When, therefore, a question affecting so large a class as the whole body of educated women comes before it, the University cannot begin to discuss the subject from the standpoint of merely conventional propriety or impropriety. To show that the kind of propriety championed by Professor Case is purely conventional it is sufficient to remember that whilst Englishmen clothe themselves from head to foot, the inhabitants of some other countries go entirely, or partially naked, and that without any sense whatever of impropriety. The old story of the American housewives who had trousers made for the legs of their tables and pianos on the ground of propriety may be commended to Mr. Case's consideration.

Sir Henry Acland is all for deciding the question of the medical education and examination of women upon the broad basis of rational principles. What conceivable objection can be made to a woman's application of a bandage to the head of an injured man? And if she may apply a bandage to his cut head, why may she not examine the wound and learn to understand it intelligently? And if it is impossible to see anything improper in her examination of the outside of the head, where does the impropriety come in if she proceeds to examine the inside and to learn all about the structure and functions of the brain? That which may be predicated of the head and the brain, may be predicated of every other part of the body. Those who believe in a Creator believe that he created the bodies of men and animals exactly as they are. To the Creator there is no doubt that every part of the human body is equally honourable with every other part. The last person in the world that we should expect to deny this would be an Oxford Professor. And why? Because we should expect an Oxford Professor to be a man of such culture, that he would be able, with the utmost facility and clearness, to distinguish between the accidental and the essential, between conventional rules and everlasting principles. These arguments are so obvious, and have been so often repeated, that we are quite ashamed even to mention them again. Practically all persons are now agreed that women may be nurses. And if they may be nurses, still more may they be doctors; because the doctor comes into much less intimate relations with the patient than does the nurse. But if a woman may be a doctor she may, nay, she must, study medicine. And if she is to study medicine, she must study it where it is taught; and if medicine is taught at Oxford, a woman may choose that University as wisely as any other. The truth is that from the standpoint of mere reason Professor Case has not a leg to stand upon. When he speaks of propriety and impropriety in this discussion he descends from the level of a University Professor and takes up the position of a Mrs. Grundy of the most illogical and prudish type.

Very wisely Sir Henry Acland declines altogether to consider the question of the propriety or impropriety of women studying medicine at Oxford, or even of their studying it along with men. These are details which must be left to the medical authorities, who are abundantly competent to deal with them. What Sir Henry Acland sees is that there is a demand on the part of women for an Oxford examination and for an Oxford testamur. That demand he wishes the University to grant. He does not see on what grounds of reason and principle the demand is to be evaded. In taking up this position he certainly has a much wiser regard for the honour of the University than that which is shown by Professor Case. Mr. Case opposes the suggested examination of women on the ground that such examination will be the insertion of the thin end of the wedge; that examination once conceded, study must follow; that if study follows, men and women may have to study together; and that such study would be improper. He even seems to think that there would be danger to morals in it. Professor Case's fears lead one to imagine that his opinion of Oxford morals is low; and that his opinion of the morals of such educated young women as would take up the study of medicine is not high. His judgment of the morality of the average Oxford student may or may not be correct; but we are convinced that so far as the morality of the average woman who chooses medicine for her profession is concerned she may study where she pleases. The danger to morals from the bringing together of men and women into the same class room is purely imaginary, and has neither reason nor experience to support it.

Few things seem to us more unchivalrous than the behaviour of men to women on this subject of medical education and examination. Instead of men welcoming women and smoothing the pathway for them, they have put every possible obstacle in their way. They have misinterpreted their motives and denied their powers. They have left women to fight their way, inch by inch, into fields of knowledge which are by right the common property of all mankind; and which, if a rational system of education prevailed, every male and female child would be made familiar with from the earliest dawn of intelligence. The proper study of mankind is man; and man in the largest sense. No university that has a regard for its honour can restrict the study of man to the phenomena of consciousness. Man cannot indeed be studied apart from his material organism. Still less can a great university declare that the study of man shall be open only to the male sex. The voice of Reason, mild-eyed and soft spoken, but unconquerable Reason, demands to be heard; and she proclaims that every creature endowed with intellect, has an indisputable claim to use that intellect on any and every subject in the heavens above, in the earth beneath, or in the waters under the earth. The business of all universities is to maintain and defend this thesis.

